# Puerperal Infection of the Urinary Tract and of the Rectum*Read before the Philadelphia Obstetrical Society, December 6, 1894.

**Published:** 1895-02

**Authors:** Richard C. Norris

**Affiliations:** Philadelphia, Obstetrician in charge of Preston Retreat, Etc.


### Selections and Abstracts.

### PUERPERAL INFECTION OF THE URINARY TRACT AND OF THE RECTUM.*

By RICHARD C. NORRIS, M. D., Philadelphia,

Obstetrician in charge of Preston Retreat, Etc.

[*From American Gynæcological and Obstetrical Journal.*]

The topic assigned me in the discussion of sepsis following labor is not so important as infection of the parturient tract since the urinary organs and the rectum are not so frequently involved in septic processes.

The literature of this subject, however, is sufficient to convince one that infection of the urinary tract is not only not uncommon, but may, indeed, be a very disastrous complication of the puerperium. Thus Schwarz noted, in 1,100 carefully observed puerperæ, catarrh of the bladder in 2.9 per cent. of the cases. The records of severe types of cystitis in childbed show a mortality of 38.8 per cent. Add to this the results of infection spreading along the ureters to the kidneys with consequent pyelitis, pyelo-nephrosis or nephritis and the sum of possible injury is well worth close attention by every one practicing obstetrics.

It will be convenient to class infection of the urinary tract as (1) *ascending* infection, *i. e.*, from the bladder or adjacent structures along the ureters to the kidneys; (2) *descending* infection—in which the kidneys are primarily, the bladder secondarily involved. Of these the former is more important because less generally recognized as a possible and dangerous puerperal complication.

Ascending infection usually begins in the bladder, the infecting poison gaining access to this viscus in one of several ways. Commonly the catheter carries the infecting agent into the bladder, either being itself not chemically clean, or if properly sterilized, but improperly introduced, it may become contaminated by decomposing lochia, at the time of its introduc-

*Read before the Philadelphia Obstetrical Society, December 6, 1894.

tion into the bladder. These dangers of the catheter can be avoided but there remains one other danger which, unfortunately, it is difficult to escape, namely, the danger of introducing into the bladder micro-organisms which are commonly found in the otherwise normal urethra. Gawronky* examined 62 women with no symptoms of urethral or cystic disease and found pathogenic bacteria in the urethra in 24 per cent. of those examined. Of ten women examined by Rovsing, bacteria were found in the urethra in six cases. Further, Rovsing† records 29 cases of cystitis, in all of which the urine contained pathogenic bacteria and as 20 of these cases followed catheterization with a clean catheter, he concludes that the cystitis was caused by the urethral microbes carried into the bladder by the catheter. Hofmeister concludes from his investigations that it is not decided whether the bladder in health contains pathogenic micro-organisms, but that the urethra is certainly rich in them. From these facts but one conclusion can be drawn, viz.: that the catheter at best is a dangerous instrument. That cystitis does not more frequently follow its use is doubtless due to the fact pointed out by Bumm, Dubelt, Rovsing and others, that a healthy uninjured bladder can resist the action of micro-organisms when introduced.

In the puerperium, however, this resisting power of the bladder is commonly not present on account of the pressure and contusion the bladder receives during labor. The experiments of Rovsing and those of Guyon are therefore of great interest and value to the obstetrician. They found that by ligating the urethra and thus causing overdistention and injury of the bladder, a catarrhal cystitis was produced, but when micro-organisms were subsequently introduced the cystitis rapidly became suppurative. These facts, then, explain why the use of the catheter is especially dangerous in puerperæ.

Ascending infection of the urinary tract may rarely occur independent of the use of the catheter. Kaltenbach first called attention to the frequency of pyelitis following labor and jointed out its connection with inflammatory processes about

*Munchener med. Wochenschrift, March 13, 1894, p. 204.

†Cystitis—its Ætiology, Pathology and Treatment, Berlin, 1890.

the uterus. Stadfeldt has also demonstrated the same affection following distention of the ureter and pelvis of the kidney, the ureter being occluded by pressure of the puerperal uterus.

Recent clinical and experimental studies of cystitis, particularly by Dogen, Clado, Halle, Albarran, Rovsing, Morelle, Denys, Schnitzler and Krogius apparently prove that micro-organisms located in any of the pelvic viscera may find their way into and infect the bladder. Reymond's* observations are especially interesting. In two cases of cystitis where the micro-organisms in the uterus and bladder were identical, treatment of the bladder was without result but after curetting and disinfecting the uterus the cystitis rapidly disappeared. In seven other cases a cure of the cystitis followed removal of the diseased pelvic organs. His four experiments upon animals showed that the introduction of organisms into the pelvis outside the bladder walls gave rise to cystitis with the micro-organisms in the bladder and not in the blood current of the pelvis. In Wreden's† experiment intestinal micro-organisms or those intentionally placed in the bowel were found in the bladder. All these observations are of value to obstetrics as an added argument for the necessity of surrounding the patient with strict cleanliness and antisepsis for they show that sepsis of the parturient tract can and does sometimes spread to the urinary organs.

Having thus briefly indicated how the bladder and ureters may become infected during the lying-in period it is easy to understand that by continuity of structure the kidneys finally are involved in the septic inflammation. Every obstetrician of wide experience will now and then see pyelitis, pyelo-nephrosis or nephritis follow infection of the bladder and ureters.

The time required for the spread of the inflammation from the bladder or adjacent structures, along the ureters to the kidneys varies. The usual time is about ten days or two weeks after the appearance of cystitis. It can however, in rare cases occur almost from the outset, before or coincident with marked bladder symptoms and in some cases the kidneys become infected only after a long-standing and persistent cystitis

*Annales des maladies des organ, genito-urinaires*, April, 1893.

†*Centralblat. f. Chirurgie*, No. 27, 1893.

or ureteritis. I have elsewhere* referred to seven cases of infected kidneys following labor and wish now to briefly report a case of ureteritis and pyelitis, at present under my care, which I believe to be the result of an infected ureter following childbirth without preceding implication of the bladder. The patient, Mrs. C., had been treated for several months for what seemed to be repeated attacks of nephritic colic. There had been frequent micturition and pain in the lumbar region since the birth of her first child four years ago. The attacks of nephritic colic first appeared when she had advanced to the sixth month of her second and last pregnancy and they increased in frequency and severity after she had been delivered. There was a history of mild infection after each of her two unassisted labors. Upon examination the right ureter was enlarged, exceedingly tender and readily palpated from its vesical orifice along the base of the bladder and upward as far as the finger could reach. The right tube and ovary were enlarged, tender and adherent. There was a tumor with distinct fluctuation in the region of the right kidney. The urine contained pus, a small amount of albumen but no casts. A perinephritic abscess was opened and drained. There was no stone, and the kidney was apparently normal. After five weeks the urinary fistula in the loin refusing to close, a No. 7 catheter was passed through the opening in the loin down the ureter to its pelvic portion but would not pass further. Vaginal examination found that the tenderness and thickening of the pelvic portion of the ureter had not disappeared and obstruction was believed to have occurred at this point, from inflammatory deposit. A ureteral bougie was passed through the opening in the loin, down the ureter into the bladder and allowed to remain three days. The bougie was thereafter passed every third day, and the ureter daily injected with boric acid solution. After two weeks of this treatment the tenderness and thickening of the ureter has almost disappeared and within a week or two the drainage tube will be removed with the expectation of cure by spontaneous closure of the opening in the loin.

That this case is not one of tubercular origin is apparen from the disappearance of all purulent discharge and inflam

*Transactions of the American Gynæcological Society, 1893.

matory deposit as well as from the negative reult of analysis of the urine for tubercle bacilli. Cases analogous to this, reported by Skene* and by Mann† have been attributed by them, to simple pressure of the child's head or of forceps during delivery, but it seems more rational, in the light of the experiment above referred to, to consider infection subequent to traumatism more important than traumatism alone.

It now remains to briefly discuss the descending form of infection of the urinary tract, *i. e.*, infection attacking the urinary organs primarily in the kidneys. This is most commonly found when there is general sepsis, metastatic abcesses occurring in the kidneys as in other organs. There are, however, cases recorded which would seem to prove that the kidneys of the puerpera, in their effort to eliminate toxic agents from the body, become infected and finally give evidence of suppurative or less marked inflammatory change such as catarrhal nephritis. The observations of Cornil, Bourget, and Griffiths‡ make it probable that the inflammatory changes result from the passage through the kidneys not only of micro-organisms themselves but also of their pathogenic products, the toxines acting upon the kidneys practically as mineral poison.

*Diagnosis.*—The early symptoms of septic puerperal cystitis are those of cystitis under other circumstances. In very severe cases exfoliation of the mucous membrane or even of the muscle walls of the bladder may occur and occasion sev ere tenesmus or retention of urine. The temperature curve will often be of value as a means to determine when the septic inflammation invades the ureters and kidneys. The fever accompanying the inflammation of the bladder is usually moderate and gradually disappears after three to six days. Should this gradual defervescence be followed, for ten days or two weeks, by an almost afebrile curve, and this by a prompt secondary rise, rapidly reaching a much greater height than had previously existed and accompanied by pain and tenderness over the kidney, the diagnosis of secondary or ascending infection of the kidneys may be assumed. Should the fever curve rise to a

---

***Transactions of the American Gynæcological Society*, 1890.

†*Ibid*, 1894.

‡Quoted by Vinas, *Traite des Maladies de la Grossesse et des suites des Couc* Paris, 1894.

great height—above 103°—at the very beginning rapid infection of the kidneys has likely occurred.

Microscopical examination of the urine also offers diagnostic signs of the extent of the inflammation, by the presence of large amounts of albumen, renal epithelium and casts. In very rare cases bacteriological examination of the urine will be necessary to determine infection of the urinary tract since it is possible to have infection without the appearance of purulent urine and without marked bladder symptoms. This is a fact to be borne in mind when those rare cases of puerperal sepsis with continued fever, rapid pulse, and extreme prostration, can not be accounted for by demonstrable local signs of sepsis along the parturient canal. It is also desirable to make repeated examinations of the urine in all cases of puerperal sepsis since it is possible for the kidneys to be coincidently involved, with either ascending or descending infection. The damage thus done to the kidneys may be very insidious and explains some of the cases of eclampsia, occurring, as late as the fourteenth or eighteenth day of the puerperium, in patients with previously healthy kidneys, Siredey has reported such a case.

*Prognosis.*—The prognosis of septic cystitis, fortunately, is favorable, provided proper treatment is begun early. Neglected cases, with ulceration and exfoliation of the bladder wall have a mortality of thirty-eight per cent., and of those who recover, the bladder, ureters and kidneys are usually permanently damaged. Pyelitis or pyelo-nephrosis is a common sequel, and chronic nephritis has thus originated. Mayer has recorded some cases ultimately dying from the kidney lesion long after their labors.

*Treatment.*—The most important part of the treatment of infection of the urinary tract after labor is its prevention. In view of the danger of introducing germs into a bladder, more or less wounded, and therefore less resistant to the action of micro-organisms, the use of the catheter after labor should be delayed as long as safety will permit and other means should be employed to secure urination. Often this will be accomplished by repeatedly placing under the patient the bedpan, filled with hot water; by the sound of running water; by assisting the patient into an upright position on her knees; by pressure over the lower portion of the abdomen, etc. While avoiding the catheter, however, the danger of over-distention of the bladder must not be forgotten, since this renders the organ more vulnerable to infecting agents. As a rule, after twelve hours, if the bladder has not been spontaneously evacuated, a chemically clean glass or soft-rubber catheter should be passed visually, having first cleansed the meatus thoroughly with a pledget of cotton and an antiseptic solution. To make assurance doubly sure it would be well, although often impracticable, to irrigate the urethra before passing the catherer into the bladder. When cystitis develops, in spite of prophylaxis, irrigation of the bladder should be carefully employed, at intervals of four hours, with mild antiseptic solutions—creolin one-half of 1 per cent. or boric acid gr. xv to the ounce. Irrigation, warm applications over the bladder and diluent drinks will usually check the disorder in a few days. When the symptoms continue and the patient becomes septic, the free use of stimulants is necessary, frequent irrigation of the bladder being meanwhile continued. When constant dribbling from the bladder in replaced by retention of urine, occlusion of the urethra by as exfoliated portion of the bladder-wall should be suspected and the separated portion should be removed, dilating the urethra for this purpose, if necessary. Before the kidneys are involved in the septic process, the administration of salol will be useful. After the kidneys were invaded in one of my cases, the daily administration of thirty grains of salol was followed by the appearance of blood in the urine so promptly that it had to be discontinued. Large doses of iron, inhalations of oxygen, and the free use of stimulants comprises the general treatment on which most reliance can be placed.

Infection of the rectum is very rare, as a primary condition; a few cases have been recorded, occurring in hospital practice, from the use of an infected syringe nozzle. Rectal infection accompanying infection of the parturient canal is not infrequently seen. Two years ago my friend Dr. Stahl consulted me about a case of sepsis following a miscarriage. The patient first developed infection of the vagina and endometrium, was douched, catheterized and was given rectal injections with the same syringe used for the vaginal douches.

She developed besides the septic endometritis and metritis, a virulent septic cystitis and septic proctitis. Pus and mucous casts of the bowel were discharged from the rectum. In spite of septic endocarditis, albuminuria and convulsions, the patient finally recovered, but with a crippled heart and with damaged kidneys.

